# Monocyte Chemotactic Protein 1 (MCP-1) Modulates Pro-Survival Signaling to Promote Progression of Head and Neck Squamous Cell Carcinoma

**DOI:** 10.1371/journal.pone.0088952

**Published:** 2014-02-19

**Authors:** Wen-Tsai Ji, Hau-Ren Chen, Chun-Hsuan Lin, Jeng-Woei Lee, Ching-Chih Lee

**Affiliations:** 1 Department of Life Science, Institute of Molecular Biology and Institute of Biomedical Science, College of Science, National Chung Cheng University, Min-Hsiung, Chia-Yi, Taiwan; 2 Department of Medical Research, Dalin Tzu Chi Hospital, Buddhist Tzu Chi Medical Foundation, Chia-Yi, Taiwan; 3 Department of Life Sciences, Tzu-Chi University, Hualien, Taiwan; 4 Institute of Medical Sciences, Tzu-Chi University, Hualien, Taiwan; 5 Department of Otolaryngology, Dalin Tzu Chi Hospital, Buddhist Tzu Chi Medical Foundation, Chia-Yi, Taiwan; 6 School of Medicine, Tzu Chi University, Hualian, Taiwan; Sun Yat-sen University Medical School, China

## Abstract

**Background:**

Monocyte chemotactic protein-1 (MCP-1) recruits monocytes and macrophages to inflammation sites, and inflammatory infiltration correlates with the progression of head and neck squamous cell carcinoma (HNSCC). This study aims to determine whether MCP-1 expression is related to HNSCC malignancy and patient survival. We also investigated the relationship between MCP-1 expression and the phosphorylation state of the pro-survival pathway factors Akt, ERK, and STAT3.

**Methods:**

Expression of MCP-1 and related proteins in HNSCC cell lines was investigated using western blotting. HNSCC patients (34) without distant metastasis at diagnosis were recruited for tissue specimen evaluation of MCP-1 expression and clinical outcomes. The relationship between MCP-1 expression and survival was evaluated using the Cox proportional hazard model with stepwise selection.

**Results:**

High-grade HNSCC cell lines were found to have higher levels of active Akt, ERK, and/or STAT3 than did lower grade cell lines under serum-free condition. OCSL, the most malignant cell line, had the highest level of endogenous MCP-1. Administration of exogenous recombinant MCP-1 increased phosphorylation of Akt, ERK, and STAT3 in a dose- and time-dependent manner and increased cellular resistance to serum starvation. Inhibition of Akt, ERK, or STAT3 reduced cell growth and caused cell death. Long-term survival of HNSCC patients was negatively associated with the histological intensity of MCP-1, implicating MCP-1 as a potential prognostic marker for HNSCC.

**Conclusions:**

These results suggest that overexpressed MCP-1 in cancer cells may promote HNSCC progression through upregulating pro-survival signaling pathways. High cellular MCP-1 expression is related to poor overall survival rate in HNSCC patients.

## Introduction

Head and neck squamous cell carcinoma (HNSCC) is the sixth most common cancer, accounting for 650 000 new cases and 350 000 deaths annually worldwide [1]. Smoking and alcohol are known risk factors for HNSCC. Infection by the human papilloma virus (HPV), especially serotype 16, has also been implicated in malignancies of the oropharynx. In South-East Asia, high rates of oral and nasopharyngeal cancer are associated with betel quid chewing and salted fish consumption, respectively [2], [3]. Despite advances in clinical therapeutics, long-term survival of HNSCC patients has not improved much over the past several decades [4]. Depending on the tumor location, consequences such as disfigurement may also seriously affect the life quality of patients after treatment [5].

Aberrantly regulated cellular signaling is known to play an important role in tumor development. Overactivation of the PI3K-Akt pathway supports cancer progression through multiple mechanisms. By activating the mTOR complex 1 (mTORC1) and downstream translation factors, Akt activity strongly enhances protein synthesis and cell proliferation [6]. Inhibition of glycogen synthase kinase 3β (GSK3β) by Akt not only results in accumulation of several oncogenic proteins, including β-catenin, c-myc, Snail, and cyclin D1 (CCND1), but also reduces expression of E-cadherin, a factor critical for contact inhibition [7]–[10]. Another effect of PI3K-Akt signaling is the downregulation of apoptosis via disabling proapoptotic proteins such as caspase 9 and Bad [11], [12]. In addition to Akt, ERK and STAT3 have been widely reported to promote tumor development. ERK and STAT3 promote proliferation, survival, and the epithelial-mesenchymal transition (EMT), as well as metastasis [13]–[15]. As in many other cancers, overactivation of Akt, ERK, and STAT3 is often observed in HNSCC. Studies of potential therapeutic agents against such pro-survival pathways are currently underway [16]–[20].

Deregulation of inflammatory processes is highly associated with cancer progression. Tumor-associated macrophages support tumor development by secreting angiogenic factors such as vascular endothelial growth factor (VEGF) [21]. Interestingly, several inflammatory cytokines secreted by tumor cells or surrounding immune cells may exacerbate carcinogenesis by upregulating pro-survival signals. Interleukin 8 (IL8) enhances Akt phosphorylation, resulting in increased synthesis of CCND1 as well as cell proliferation [22]. PI3K-Akt signaling also mediates the effects of IL6 on cell growth and survival [23]. Because of the positive correlation between inflammation and tumor development, increased serum levels of the inflammatory mediators IL6 and IL8 have been suggested as potential prognostic indicators of cancer progression [24]–[26].

Monocyte chemotactic protein 1 (MCP-1/CCL2) is a 76–amino-acid peptide secreted by fibroblasts, endothelial and epithelial cells, monocytes, and various tumor cells. Important roles of MCP-1 include the recruitment of monocytes and macrophages into sites of inflammation and the regulation of their activities [27]. Accumulating evidence suggests that MCP-1 may also influence cancer malignancy [28]–[31]. Although inflammation has been shown to correlate with HNSCC progression, the precise role of MCP-1 in the progression of this disease remains obscure [32].

The aim of this study was to determine whether MCP-1 expression is related to the grade of HNSCC malignancy and patient survival. We also investigated the relationship between MCP-1 expression and the phosphorylation state of the pro-survival pathway factors Akt, ERK, and STAT3.

## Materials and Methods

### Ethics Statement

This study was initiated after being approved by the Institutional Review Board of the Buddhist Dalin Tzu Chi General Hospital, Taiwan. Review board requirements for written informed consent in immunohistochemical staining and survival analysis were waived because all personal identifying information was removed from the database prior to data analysis.

### Cell Culture

Fadu, a hypopharynx squamous cell carcinoma cell line, and 2 oral cancer cell lines, OC2 and OCSL, two oral squamous cell carcinoma cell lines derived from two Taiwanese men with habits of drinking, smoking, and areca nut chewing [33], were maintained in RPMI medium supplemented with 10% fetal bovine serum (FBS) and 1% penicillin/streptomycin. The oral cancer cell lines SCC4 and SCC25, two tongue squamous cell carcinoma cell lines, were maintained in DMEM/F12 with similar supplements. Cells were routinely kept in a 37°C incubator supplied with 5% CO_2_ and subcultured every 2 to 3 days. 12 to 16 hours after seeding, experiments were performed soon after medium refreshing, when cell confluence was about 70–90% except for viability tests (20–30% confluence). Fadu, SCC4 and SCC25 were obtained from ATCC. For serum starvation, cells were washed twice with Phosphate buffered saline (PBS) and cultured in serum-free medium immediately before experiments. For PI3K inhibitor experiments, cell were treated with or without Ly294002 (a PI3 Kinase inhibitor) and then treated MCP-1.

### Reagents and Antibodies

Dimethyl sulfoxide (DMSO) was purchased from Sigma-Aldrich (St. Louis, MO, USA). The antibody against survivin was purchased from Cell Signaling Technology (Danvers, MA, USA). PI3 Kinase inhibitor Ly294002 was purchased from Calbiochem (San Diego, CA). The antibody against cleaved PARP (24 kDa) was purchased from Epitomics (Burlingame, CA, USA). Antibodies against fibronectin, snail, dysadherin, STAT3, phosphorylated STAT3 (Y705), vimentin, and MCP-1 were purchased from Abcam (Cambridge, UK). The antibody against E-cadherin was purchased from BD Biosciences (San Jose, CA, USA).

### Cell Lysate Preparation and Western Blot Analysis

Cells in 24-well plates were washed twice with PBS and lysed with 70 µL 4X Laemmli loading buffer, followed by boiling for 10 minutes. Equal amounts of samples were run on SDS-PAGE gels and transferred to PVDF membranes. Expression of individual proteins was detected using corresponding antibodies, followed by the secondary antibody conjugated with horseradish peroxidase (HRP). After incubation with enhanced chemiluminescence (ECL), the membranes were exposed to X-ray film (Kodak).

### Viability Analysis

Cells at 70% confluence were treated with the indicated reagents. One day later, MTT reagent (Sigma) was added to each well to a final concentration of 1 mg/mL. Plates were swirled gently for a few seconds and cultured continuously for 3 hours. After incubation, the medium was removed. Cells were washed twice with PBS, and the MTT metabolic product was resuspended in 500 µL DMSO. After swirling for seconds, 50 µL supernatant from each well was transferred to optical plates for detection at 595 nm.

### Immunohistochemical Staining

This study included 34 patients with human head and neck squamous cell carcinoma without distant metastasis, newly diagnosed between 2000 and 2009. All of these patients initially underwent surgical curative treatment and, with the approval of our IRB, had tissue samples available for immunohistochemical staining. The following data were collected: patient age, gender, pathological TNM stage, treatment modality, and outcomes (local/regional recurrence, distant metastasis, and death). All tumors were staged according to the American Joint Committee on Cancer (AJCC) cancer system modified in 2002.

Sample processing and immunohistochemical staining were performed as previously described [34]. MCP-1 expression was detected immunohistochemically in 34 human head and neck squamous cell carcinoma tissues. Paraffin sections were deparaffinized with xylene, rehydrated, submerged in citrate buffer, microwaved for antigen retrieval, and treated with 3% hydrogen peroxide in methanol to quench endogenous peroxidase activity. Sections were then washed in 1% BSA, treated with mouse monoclonal anti MCP-1 antibody (1∶100X, ab9858; AbCam, Cambridge, United Kingdom) overnight at 4°C, incubated with a secondary antibody from the UltraVision Quanto Detection System HRP DAB system (Thermo Fisher Scientific, CA, United Kingdom) at room temperature, and subsequently visualized by reaction with diaminobenzidine (DAB) used as the chromogen substrate. Negative controls were obtained by omitting the primary antibody. Two independent observers assessed MCP-1 protein expression. The percentage of positive tumor cells was determined and graded (from 0–5): 0% (0), 1–20% (1), 21–40% (2), 41–60% (3), 61–80% (4), and >81% (5). The MCP-1 intensity was scored as negative (0), mild (1), moderate (2), or intense (3) at 100×magnification. The two parameters were combined (intensity score (from 0–3)+the grad of positive tumor cells percentage (from 0–5)), resulting in an overall score of 0–8. The scores were classified into three groups: group 1 (score 0, no expression); group 2 (score 2–4, low expression); and group 3 (score ≧ 5, high expression).The scoring method was performed as previously described [35].

### Statistical Analysis

All data were analyzed using the SPSS system (version 15, SPSS Inc., Chicago, IL, USA). The association between the categorical variables was analyzed using Pearson’s chi-square test or Fisher’s exact test. Continuous variables were compared using one-way ANOVA. Survival rates at different levels of cellular MCP-1 expression were compared using Kaplan-Meier curves and the log-rank test. The Cox proportional hazard model with stepwise selection was used to find the independent variables for cancer outcomes. The data were analyzed using the t-test; results with p-values less than 0.05 were defined as significant.

## Results

### Activation of Akt, ERK, and STAT3 in High-grade HNSCC Cells

**Figure 1:**
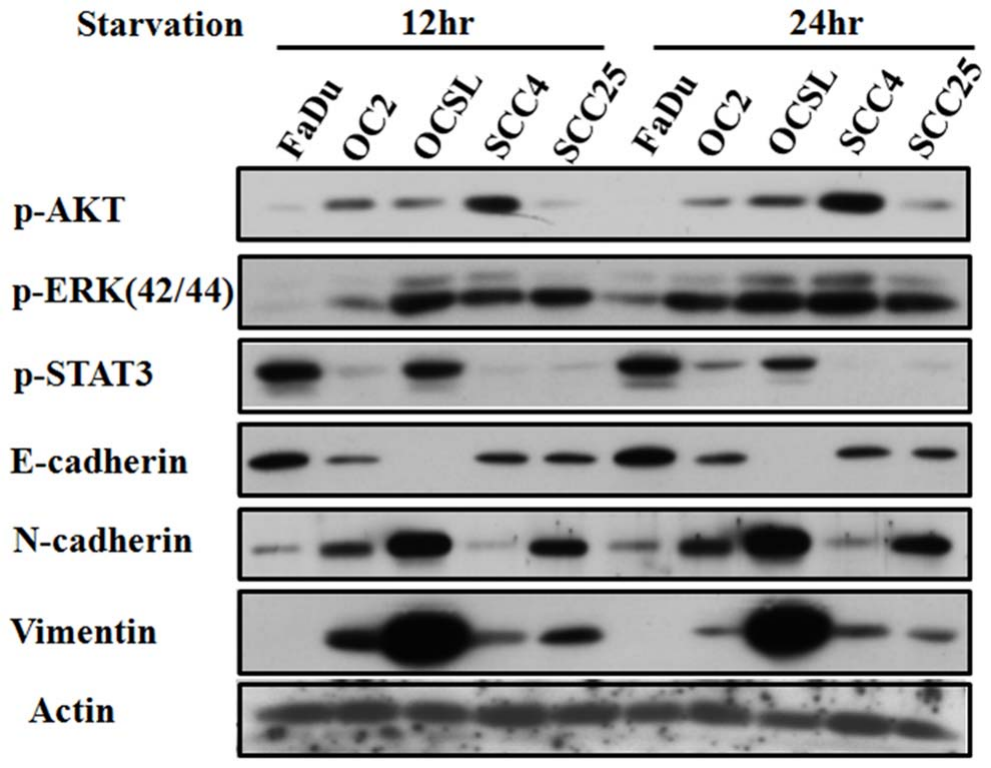
Correlation between cell malignancy and the activities of Akt, ERK, and STAT3. The cell malignancy sequence was OCSL, OC2, SCC25, SCC4, and Fadu according to the mesenchymal marker expression of epithelial-mesenchymal transition. FaDu was as least malignant and OCSL was as the most malignant.

In general, the high-grade HNSCC cell lines (particularly the OCSL cells) had higher levels of phosphorylation of active Akt, ERK, and STAT3 (Fig. 1). Accordingly, low-grade cells (such as Fadu) had lower levels of phosphorylated pro-survival factors except STAT3. These results suggest that aggressive HNSCC cells have a stronger endogenous ability to maintain Akt, ERK, and STAT3 signaling under serum-free condition.

### Upregulation of Akt, ERK, and STAT3 Activity by MCP1

**Figure 2:**
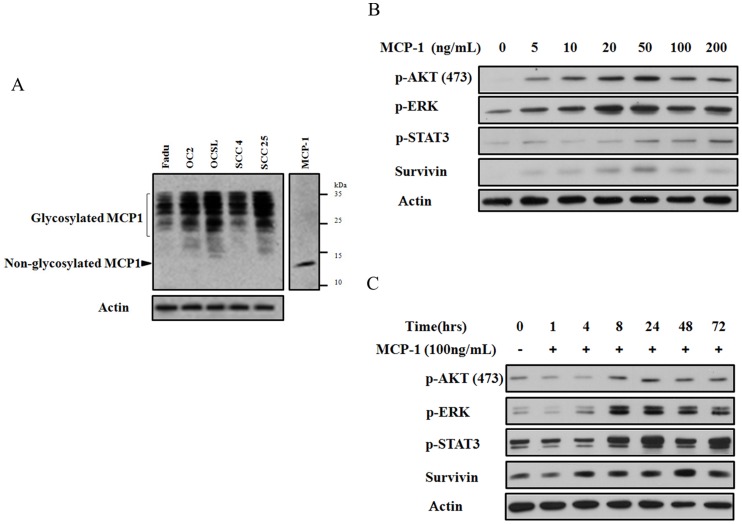
Upregulation of Akt, ERK, and STAT3 phosphorylation by MCP1. (A) Endogenous expression of MCP-1 was evaluated in different HNSCC cell lines under serum-free condition. (B) Recombinant MCP-1 was used to treat Fadu cells. MCP-1 treatment enhanced phosphorylation of Akt, ERK, and STAT3 and increased their survival in a dose-dependent manner. (C) MCP-1 treatment enhanced phosphorylation of Akt, ERK, and STAT3 and increased their survival in a time-dependent manner.

Since high-grade HNSCC cells have highly active pro-survival signaling, we further examined whether MCP-1 is a key factor in maintaining the phosphorylation of these factors. To elucidate the role of MCP-1 in tumor progression, endogenous expression of MCP-1 was also evaluated in different HNSCC cell lines under serum-free condition. Interestingly, the most malignant cell line, OCSL, had the highest level of MCP1, suggesting the involvement of MCP-1 in HNSCC progression (Fig. 2A). Furthermore, recombinant MCP-1 was used to treat Fadu cells, a cell line with less endogenous MCP-1 (Fig. 2A). As shown in Fig. 2B and 2C, MCP-1 treatment enhanced phosphorylation of Akt, ERK, and STAT3 and increased their survival in a dose- and time-dependent manner. However, cells treated with doses of MCP-1 over 50 ng/mL did not survive well and did not undergo further increases in Akt/ERK phosphorylation and survivin expression, suggesting that overdoses of MCP-1 might exert feedback inhibition. Similar effects were observed in stable cells with ectopically expressed MCP-1, although Akt phosphorylation was similarly downregulated (data not shown). These results suggest that endogenous MCP-1 might support pro-survival signaling in HNSCC cells under serum-free condition.

### MCP-1 Increases Resistance to Serum Deprivation

**Figure 3:**
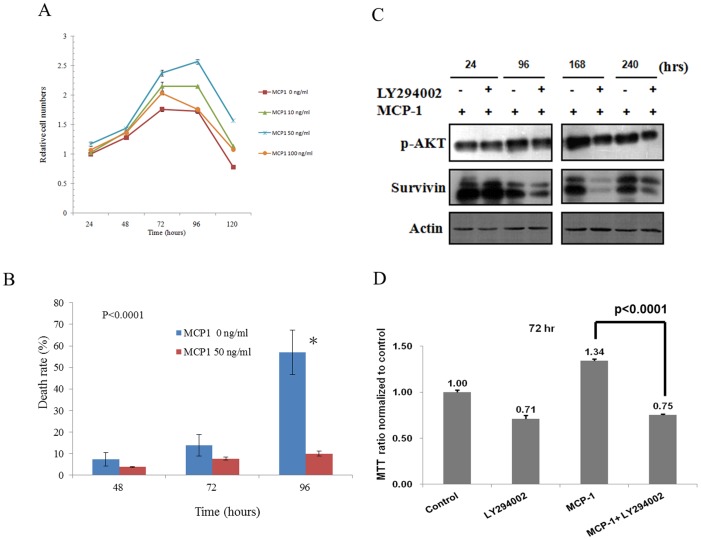
Increase in resistance to serum starvation by MCP-1. (A) Administration of MCP-1 detectably increased cell growth under serum-free condition in MTT assay. (B) Administration of MCP-1 detectably increased cell viability under serum-free condition. (C) Inhibition of PI3K-Akt signaling by LY294002 reduced survivin expression. Fadu cells were pretreated with or without PI3K inhibitor LY294002 20 uM for 1 hour, and then treated with 50 ng MCP-1 for indicated times. Cells were harvested, and subject to Western blot. (D) Inhibition of PI3K-Akt signaling by LY294002 antagonized the effect of MCP-1 on cell survival. Fadu cells were pretreated with or without PI3K inhibitor LY294002 50 uM for 1 hour, and then treated with or without 50 ng MCP-1 for 72 hours. Cells were subject to MTT assay. Control : Fadu cells were treated without LY294002 and MCP-1 for 72 hours.

Because Akt, ERK, and STAT3 were clearly shown to be associated with cell survival and proliferation, the effects of MCP-1 and pro-survival signaling on cellular growth and survival were investigated. As expected, administration of MCP-1 detectably increased cell growth and viability under serum-free condition (Fig. 3A, 3B). Consistent with the phosphorylation status of pro-survival factors, MCP-1 at 50 ng/mL exerted the strongest effects on cell proliferation. In contrast, inhibition of PI3K-Akt signaling by LY294002 significantly reduced survivin expression and antagonized the effect of MCP-1 on cell survival (Fig. 3C, 3D). These results suggest that MCP-1 might increase cellular resistance against environmental stresses such as serum starvation and thus facilitate the progression of HNSCC.

### Clinical Outcomes

**Figure 4:**
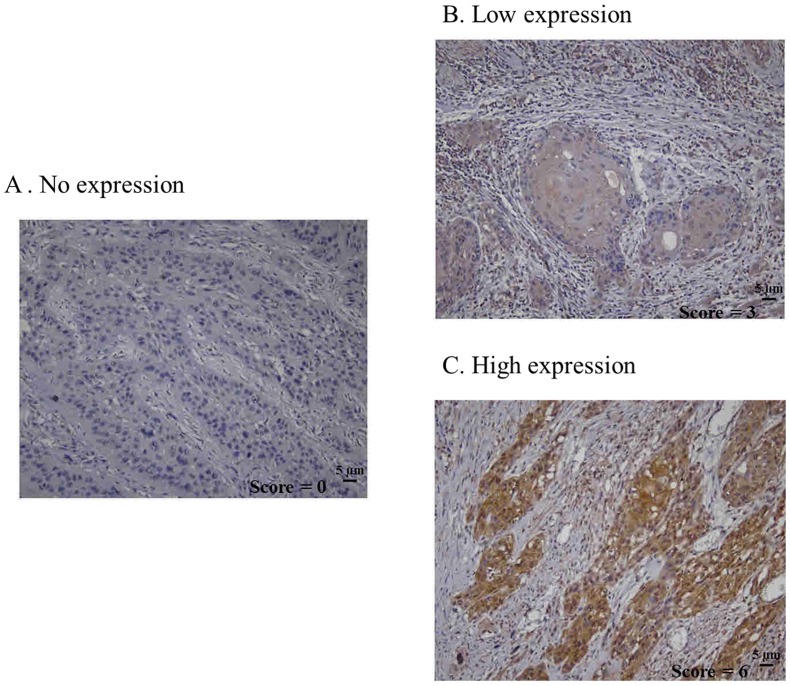
Expression of MCP-1 in head and neck squamous cell carcinoma cancer tissues. (A) No expression. (B) Low expression. (C) High expression (×100).

**Figure 5:**
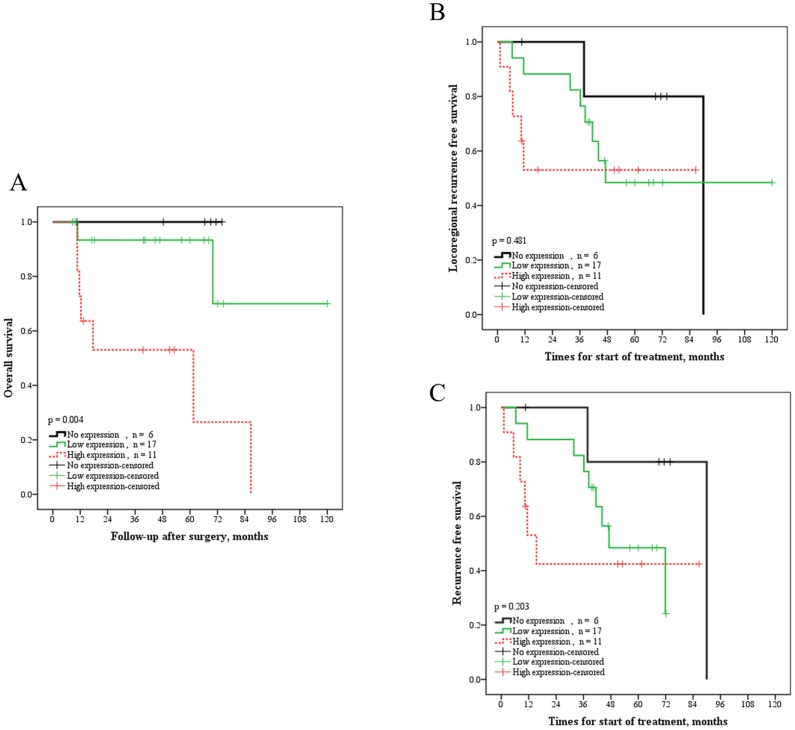
Correlation between the histological level of MCP-1 and patient long-term survival in head and neck squamous cell carcinoma cancer. (A) Overall survival in HNSCC patients with different levels of MCP-1expression. (B) Locoregional recurrence in HNSCC patients with different levels of MCP-1expression. (C) Any recurrence in HNSCC patients with different levels of MCP-1expression.

Of the 34 patients, 17 (50%) relapsed within a median follow-up of 48 months (range, 9–120 months). Of the relapsed patients, 15 (44%) had locoregional recurrence (local recurrence in primary tumor site or regional recurrence in cervical lymph nodes) and 2 (6%) had distant metastasis. Nine (26%) patients died in this series. Table 1 shows the association of clinical characteristics and MCP-1 IHC. A high IHC score was associated with advanced pN classification (P = 0.003) and advanced pathological stage (P = 0.007). Figure 4A, 4B, 4C, 5A, 5B and 5C depict the survival curves for overall survival, locoregional recurrence, and any recurrence. Patients with high IHC incurred poor overall survival (P = 0.004). Using the Cox proportional model with stepwise selection, high MCP-1 expression was a significantly negative predictor for overall survival (HR, 9.37; 95% CI, 1.88–47) (Table 2).

**Table 1:** Baseline characteristics of human head and neck squamous cell carcinoma patients and immunohistochemical staining (IHC).

	Number of patients (%)		IHC score			p-value
			No expression	Low expression	High expression	
Age of patients (mean, SD)			58±13	55±12	49±10	0.259
Gender						0.283
Female	2	(6)	1	0	1	
Male	32	(94)	5	17	10	
pT stage						0.369
1–2	26	(78)	6	12	8	
3–4	7	(22)	0	4	3	
pN stage						0.003
0–1	28	(85)	6	16	6	
2–3	5	(15)	0	0	5	
pAJCC stage						0.007
I–II	24	(70)	6	14	4	
III–IV	10	(30)	0	3	7	
Treatment						0.063
Surgery alone	14	(41)	5	6	3	
Surgery +adjuvant treatment	20	(59)	1	11	8	

Values are given as number (percentage).

**Table 2:** Factors associated with disease-specific survival in human head and neck squamous cell carcinoma patients.

	Locoregional recurrence		Any recurrence		Overall recurrence	
	Univariate	Adjusted HR*	Univariate	Adjusted HR	Univariate	Adjusted HR
		(stepwise selection)		(stepwise selection)		(stepwise selection)
Age	0.98		0.98		0.92	
	(0.93–1.03)		(0.94–1.03)		(0.85–0.99)	
Female gender	0.05		0.9		1.61	
	(0.00–887.46)		(0.12–6.95)		(0.20–13.27)	
pAJCC stage III/IV	2.09		3.3		3.11	
	(0.73–5.99)		(1.23–8.87)		(0.81–11.91)	
Surgery+adjuvant treatment	4.42	4.42	7.65	7.65	2.65	
	(1.23–15.89)	(1.23–15.89)	(1.72–34.03)	(1.72–34.03)	(0.53–13.34)	
High expression IHC score	1.78		1.98		9.37	9.37
	(0.59–5.37)		(0.71–5.50)		(1.88–46.74)	(1.88–46.74)

Values are given as HR (95% confidence interval).

*Adjusted HR, Adjusted Hazard ratio. Values are given as Adjusted HR (95% confidence interval).

## Discussion

High-grade HNSCC cell lines were found to have higher levels of active Akt, ERK, and/or STAT3 than did lower grade HNSCC cell lines under serum-free conditions. The most malignant cell line, OCSL, had the highest level of endogenous MCP-1. Moreover, administration of exogenous recombinant MCP-1 increased phosphorylation of the pro-survival factors Akt, ERK, and STAT3 in a dose- and time-dependent manner. Treatment with exogenous MCP-1 also increased cellular resistance against serum starvation stress. Inhibition of Akt, ERK, or STAT3 clearly reduced cell growth and caused cell death, especially under serum-free conditions. Long-term survival of HNSCC patients was negatively associated with the histological intensity of MCP-1.

Overall, our results indicate that MCP1 may enhance HNSCC progression via upregulation of pro-survival signaling involving Akt, ERK, and/or STAT3. Within the tumor microenvironment, activation of pro-survival signaling can be mediated by cytokines released from surrounding cells. Our results showed that HNSCC cell lines maintained phosphorylation of Akt, ERK, and STAT3 under serum free condition and expressed more MCP-1, even though the Fadu cell line has highly active STAT3. Since recombinant MCP-1 significantly upregulated pro-survival signaling, these results contribute to the evidence that endogenous MCP-1 enhances cancer malignancy and survival independent of tumor-associated cells, although a few benign cell lines like HaCaT were reported to have many MCP-1 transcripts [36].

The detailed mechanism whereby MCP-1 simultaneously activates pro-survival signals in HNSCC cells remains unclear. IL6, EGF, and leukemia inhibitory factor (LIF) have been shown to induce the simultaneous phosphorylation of Akt, ERK, and STAT3; thus, the mediators through which MCP-1 upregulates pro-survival signaling may be elucidated in the near future [37]–[39]. Nevertheless, the possibility that MCP-1 regulates these factors through a mechanism independent of IL6/EGF/LIF should not be ruled out.

Under our conditions, a concentration of 50 ng/mL MCP-1 was found to be the threshold for strong activation of pro-survival signaling at 24 hours after treatment. Interestingly, an overdose of MCP-1 did not further enhance but rather inhibited the effects on Akt and survival, particularly in stably transfected cells (data not shown). Higher doses of MCP-1 might induce activation earlier, resulting in a decline in the phosphorylation of pro-survival factors at the observed time point; however, it seems likely that the high dose of MCP-1 indeed caused negative effects in HNSCC cells, as 100 ng/mL of MCP-1 was also less effective in supporting cell growth under serum starvation condition (Fig. 3A). A possible explanation for this observation is that an overdose of MCP-1 might induce negative feedback inhibition, as suggested in a previous study [40]. Although an *in* *vitro* overdose of exogenous MCP-1 might downregulate pro-survival signaling, it should be noted that the *in* *vivo* histological concentration of extracellular MCP-1 within cancer tissues might not be as high as that administrated *in* *vitro*.

We observed that high histological intensity of MCP-1 correlated positively with poor long-term survival of HNSCC patients. Due to the diversity of HNSCC, histological biomarkers may be necessary for assessing cellular malignancy and metastatic potential, thus allowing minimization of aggressive treatments that have serious consequences like disfigurement. Inflammation is associated with cancer development and the infiltration of immune cells, leading some to suggest the presence of macrophages as a marker of poor prognosis. However, evaluating cancer severity by quantifying the degree of infiltration may be difficult. Theoretically, the histological intensity of MCP-1 could be used to assess cancer malignancy, although tumor-associated cells might also contribute to the accumulation of MCP-1 [41]. In breast cancer, the concentration of MCP-1 in tissue extracts has been similarly reported as an indicator of early relapse, further supporting the possibility of using histological MCP-1 to determine the aggressiveness of HNSCC [42].

There are several limitations to our study. First, we did not determine the effects of abolishing MCP-1 activity using MCP-1 siRNA or anti-MCP-1 antibodies. Second, the number of patients clinically evaluated was small. However, using the stepwise selection method, MCP-1 was an independent prognostic factor for overall survival. Third, the pathway downstream of STAT3 was not evaluated.

Taken together, our results suggest MCP-1 may enhance HNSCC progress via upregulation of pro-survival signaling. MCP-1 was an independent prognostic factor for overall survival using clinical data. The close correlation between MCP-1 and long-term survival implicates the histological intensity of this protein as a potential prognostic biomarker for HNSCC.
